# Ubiquitination mediated by RING-type E3 ligases in the progression of digestive system tumors: mechanistic insights and potential therapeutic strategies

**DOI:** 10.3389/fcell.2026.1809222

**Published:** 2026-05-20

**Authors:** Yuwei Wu, Xi Yu, Chao Guan, Mingfang Zhao

**Affiliations:** 1 Center for Cell and Gene Therapy, The First Hospital of China Medical University, Shenyang, China; 2 Editorial Office of Journal of China Medical University, Journal Center of China Medical University, Shenyang, China; 3 Department of Oncology, Shengjing Hospital of China Medical University, Shenyang, China; 4 Department of Medical Oncology, The First Hospital of China Medical University, Shenyang, China

**Keywords:** digestive system tumors, drug resistance, PTMs, RING-type E3 ligase, ubiquitination

## Abstract

Ubiquitination is a core mechanism for the precise regulation of protein fate, closely related to the occurrence and development of many diseases. As the most critical enzymes in the ubiquitination process, really interesting new gene (RING)-type E3 ubiquitin ligases play an important role in regulating the malignant behaviors of tumor cells. As tumor promoters or suppressors, different types of RING-type E3 ligases have shown various prognostic values in cancer patients. This review provides a comprehensive overview of the effects of RING-type E3 ligases on the progression of digestive system tumors, highlighting the key signaling pathways involved in regulating tumor proliferation, invasion, migration, and metastasis. Additionally, it discusses the relationship between the expression of RING-type E3 ligases and poor patient prognosis, providing a foundation for clinical screening of valuable prognostic markers. Furthermore, this review summarizes recent research progress, mechanisms of action, and current challenges related to anti-tumor small molecule inhibitors targeting various classes of RING-type E3 ligases. Further studies on the mechanisms of RING-type E3 ligases in the treatment of digestive system tumors will aid in identifying new targets and provide theoretical support and research strategies for developing anti-tumor drugs.

## 1 Introduction

According to the latest global cancer statistics, the incidence and mortality rates of digestive system tumors are among the highest, making them a significant public health problem affecting human health. The five major digestive system tumors (colorectal, gastric, liver, esophageal and pancreatic cancers) account for 23.6% of all new global cancer cases and 33.6% of all global cancer-related deaths (Bray et al., 2024). The malignant progression and distant metastasis of tumor is a complex pathological process regulated by multiple steps and multiple factors. The core of the process mainly includes the abnormal proliferation of tumor cells, local invasion, directional migration and distant organ colonization. Therefore, behaviors such as proliferation, invasion, and migration, together with malignant phenotypes such as immune evasion and treatment resistance, constitute the complete biological characteristics of tumor progression, jointly promoting the deterioration and metastasis of the disease. In this cascade, immune escape, as the core malignant phenotype throughout the whole process of tumor development, is interwoven and synergistically promoted with biological behaviors such as cell proliferation, invasion and migration (Roerden and Spranger, 2025). Tumor cells evade immune surveillance and immune clearance through a series of mechanisms, which can not only maintain their own continuous proliferation, but also further enhance local invasion ability, promote vascular invasion and perineural invasion, and resist immune killing in the circulatory system and the distant microenvironment, which ultimately lay the foundation for successful completion of distant metastasis (Galassi et al., 2024). Therefore, behaviors such as proliferation, invasion and migration together with malignant phenotypes such as immune escape and treatment resistance constitute the complete biological characteristics of tumor progression, which jointly promote the deterioration and metastasis of the disease. Currently, treatments for digestive system tumors cannot fundamentally prevent drug resistance or the progression of advanced tumors (Schoenfeld and Hellmann, 2020; Vasan et al., 2019; Carneiro and El-Deiry, 2020; Morad et al., 2021; Marusyk et al., 2020). The main causes of malignant tumor progression include genetic or epigenetic changes, changes in stemness characteristics, abnormal regulation of post-translational protein modifications (PTMs), immunosuppression within the tumor microenvironment, adaptive reprogramming of metabolic pathways, and individual differences among patients (de Visser and Joyce, 2023). PTMs act as “molecular switches” at different stages of tumor development by precisely controlling protein activity. Targeting PTMs-related enzymes (such as kinases, acetyltransferases, ubiquitinases, glycosyltransferases, etc.) and modification sites has become a necessary research focus for precise tumor therapy (Vu et al., 2018). Common types of PTMs include phosphorylation, glycosylation, ubiquitination, acetylation, methylation, and SUMOylation. These modifications precisely regulate protein expression through the dynamic equilibrium of enzymatic reactions, forming a complex regulatory network of protein functions.

The ubiquitin-proteasome system (UPS) is essential for regulating and maintaining cellular homeostasis, and is an important pathway for protein degradation in eukaryotic cells (Deng et al., 2020). Ubiquitination is a key type of post-translational modification of proteins, involving a three-enzyme cascade that includes ubiquitin (Ub), ubiquitin-activating enzyme (E1), ubiquitin-conjugating enzyme (E2), and ubiquitin-ligating enzyme (E3). Among these, the E3 ubiquitin ligase serves as the primary regulator of UPS specificity and efficiency. Eukaryotic E3 ligases are divided into three families based on their structural and functional domains: really interesting new gene (RING) type E3 ubiquitin ligases, homologous to E6-AP carboxyl terminus (HECT) type E3 ubiquitin ligases, and ring between ring fingers (RBR) protein family. There are over 30 types of HECT E3 ligases, and the HECT domain forms a thioester bond (E3∼Ub intermediate) with the ubiquitin carried by E2s, transferring the ubiquitin to the substrate (Rotin and Kumar, 2009; Huang et al., 1999). The RBR family, a minor subgroup of RING finger E3 ligases, consists of only 14 known human members E3 ligases includes three domains: RING1 (which binds E2), an in-between-RING (IBR) domain, and RING2 (which has a catalytic cysteine residue) (Spratt et al., 2014). In contrast, the RING-type E3 ligases make up the largest group, with over 600 members. They directly enable E2 to bind to the substrate without creating a covalent E3–Ub intermediate (Deshaies and Joazeiro, 2009; Li et al., 2008). Because of their simple mechanism and abundance (comprising over 90% of E3 ligases), RING-type E3 ligases are the primary focus of research in various disease areas.

This review emphasizes the crucial roles of RING-type E3 ubiquitin ligases in the progression and drug resistance of digestive system tumors. It also summarizes current anti-tumor therapies targeting these ligases, aiming to support future drug development and offer new insights for clinical diagnosis and treatment.

## 2 Classification and function of RING-type E3 ligases

**FIGURE 1:**
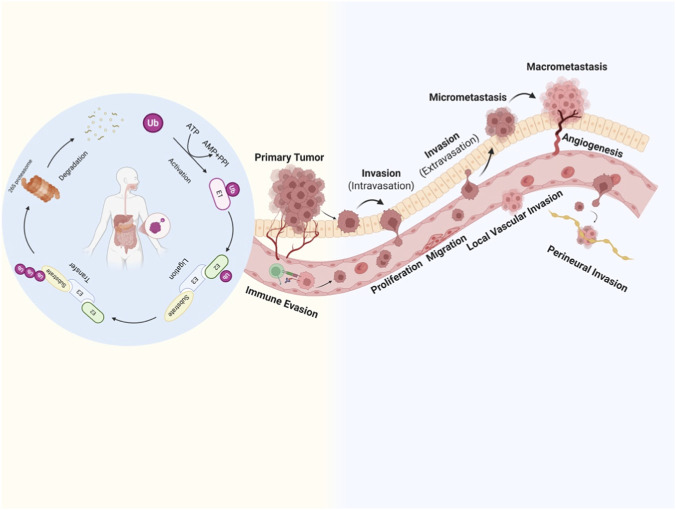
Integrating the ubiquitination process into the four stages of tumor progression helps us better understand the mechanism of tumor metastasis. Left panel: The core enzymatic cascade of the UPS, including E1 ubiquitin-activating enzyme, E2 ubiquitin-conjugating enzyme, and E3 ubiquitin ligase, mediates the covalent attachment of ubiquitin (Ub) to substrate proteins, targeting them for degradation by the 26S proteasome. This process is tightly regulated and can be pharmacologically modulated by proteasome inhibitors (PPI). Right panel: The UPS is critically involved in every step of tumor progression, from primary tumor growth to distant metastasis. Key processes depicted include: tumor cell proliferation, immune evasion, local invasion (intravasation), migration, local vascular invasion, perineural invasion, extravasation, micrometastasis, macrometastasis, and angiogenesis. Dysregulation of the UPS promotes these malignant phenotypes, thereby accelerating disease progression and metastasis in digestive system malignancies.

Ubiquitination is a multi-step enzymatic process in which Ub, a protein composed of 76 amino acids, becomes conjugated to substrate proteins. In the process of ubiquitination, three enzyme classes: E1 (activating enzyme), E2 (conjugating enzyme), and E3 (ubiquitin ligase) cooperate in a stepwise manner to complete the activation of ubiquitin and the labeling of target proteins. This process involves three main steps: activation, transfer, and ligation. E1 activates ubiquitin, forming an “E1-ubiquitin” complex, and then the activated ubiquitin is transferred from E1 to the cysteine residue of E2. Finally, E3 recognizes specific target proteins and catalyzes the transfer of ubiquitin from E2 to the lysine residue on the substrate, completing the conjugation (Sun et al., 2020; Zhang Z. et al., 2023) (Figure 1). As a key part of the UPS, RING-type E3 ubiquitin ligases are defined by the presence of a RING domain. This structural feature is stabilized by zinc ions (Zn^2+^) coordinated by conserved cysteine and histidine residues. The RING domain acts as a docking platform for E2-ubiquitin conjugates and is crucial for facilitating the efficient transfer of ubiquitin to the target protein (Berndsen and Wolberger, 2014; Jackson et al., 2000; Deshaies and Joazeiro, 2009).

The composition and structural features of RING-type E3 ligase subunits can be classified into three subtypes: monomeric, heterodimeric, and multisubunit RING finger E3 ligases (Sampson et al., 2023). Monomeric RING finger E3 ligases include tripartite motif proteins (TRIM), RING finger protein family (RNF), and TNF receptor-associated factors (TRAF), cellular inhibitor of apoptosis protein (cIAP), X-linked inhibitor of apoptosis protein (XIAP) (Campbell et al., 2012; Fiorentini et al., 2020; Mace et al., 2008; Nakatani et al., 2013). Heterodimeric RING finger E3 ligases include complexes such as murine double minute 2/4 (MDM2/4) and breast cancer type 1 susceptibility protein/breast cancer-associated RING domain protein 1 (BRCA1/BARD1) (Linke et al., 2008; Brzovic et al., 2001). Cullin ring finger ligase (CRL) and anaphase-promoting complex/cyclosome (APC/C) are both multisubunit ring finger E3 ligases (Zhao and Sun, 2013; Schrock et al., 2020).

Among these E3 ligases, TRIMs are a group of proteins that contain conserved domains like the RING finger, B-box, and Coiled-Coil domains. They have E3 ubiquitin ligase activity and play important roles in various biological and disease-related processes, such as antiviral immunity, cell differentiation, tumor regulation, and maintaining protein balance—through substrate ubiquitination (Hatakeyama, 2017; Ozato et al., 2008; Aizaz et al., 2023; Lu et al., 2022). The RNF family is a group of proteins characterized by the RING finger domain, named for its “finger-like” structure. These proteins facilitate ubiquitination and the degradation of substrates. Acting as proto-oncogenes or tumor suppressors, RNF proteins are involved in regulating cell proliferation and differentiation in cancer (Lipkowitz and Weissman, 2011; Koo et al., 2012). The domains of the TRAF family proteins typically include the N-terminal RING domains, zinc finger domains, and C-terminal TRAF domains. They have E3 ubiquitin ligase activity and facilitate protein ubiquitination. The TRAF protein family is involved in immune response, inflammatory response, and cell apoptosis by regulating signaling pathways such as NF-κB and mitogen-activated protein kinase (MAPK) (Siegmund et al., 2022; Borghi et al., 2016; Vince et al., 2009). MDM2/4 proteins have multiple functional domains: p53-binding regions and zinc finger structures at the N-terminus, and RING finger domains at the C-terminus. MDM2/4 promotes the ubiquitination and degradation of p53, thus supporting the development and progression of various tumors (Haupt et al., 1997; Lenos and Jochemsen, 2011). The S phase kinase-associated protein 1–cullin1–F-box protein (SCF) E3 ligases represent the largest group within the CRL family. The SCF-type E3 ligase is a multi-subunit RING complex composed of S-phase kinase-associated protein 1 (SKP1), Cullin 1, RING-box protein 1 (RBX1), and a variable F-box protein. It precisely regulates substrate degradation and signal transduction through ubiquitination, participating in pathophysiological processes such as cell cycle progression and deoxyribonucleic acid (DNA) repair (Galindo-Moreno et al., 2019; Lu et al., 2012). SKP1 serves as an adaptor protein, recruiting F-box proteins into the SCF complex. Cullin 1 acts as the structural scaffold, maintaining the stability of the SCF complex. *RBX1* has a RING domain that helps transfer ubiquitin from E2 enzymes to substrate proteins. The F-box protein determines the substrate specificity of the SCF complex, with different F-box proteins recognizing various substrates. F-box proteins have been confirmed to play roles in cell cycle regulation, cell differentiation, cell apoptosis, and signal transduction, and are currently considered promising targets for drug development (Kipreos and Pagano, 2000; Jin et al., 2004).

In view of the functional heterogeneity and substrate specificity among different families of RING-type E3 ligases, we have determined their mechanism of action in the development of digestive system tumors and established a theoretical basis for targeted therapy approaches. By clarifying the roles and molecular features of E3 ligases in tumor initiation and progression, we aim to identify key target proteins. Additionally, we provide concise summaries and prospects for the research and development of small-molecule anti-tumor inhibitors.

## 3 The mechanism of RING-Type E3 ligases in different digestive system tumors

The development of digestive system tumors is insidious and prone to metastasis. However, the drugs or methods for treating advanced tumors are limited and challenging. Current research indicates that RING-type E3 ligases not only participate in the progression of digestive system tumors but also serve as prognostic biomarkers for predicting tumor behavior. Therefore, RING-type E3 ligases may be promising biomarkers and prognostic indicators, offering valuable reference for diagnosis and treatment strategies (Supplementary Table 1).

### 3.1 Colorectal cancer

#### 3.1.1 TRIM family

**FIGURE 2:**
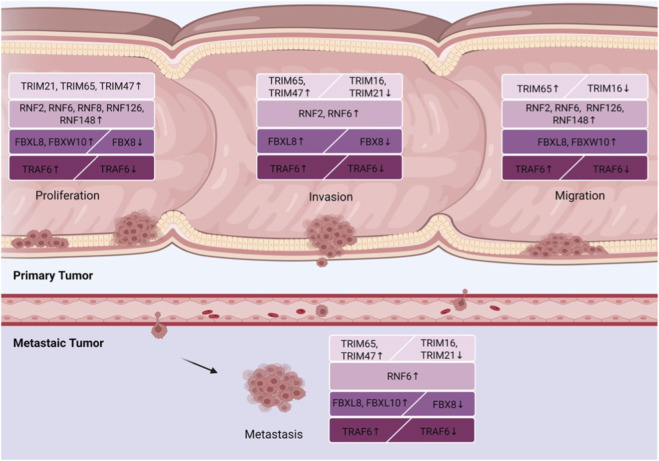
The roles of RING-type E3 ligases in the four processes involved in CRC progression. Among them, ↑ indicates a promoting effect on CRC progression, and ↓ indicates an inhibitory effect on CRC progression.

As a core subfamily of RING-type E3 ligases, the TRIM family governs key oncogenic signaling cascades, and its dysregulation critically drives colorectal cancer progression and clinical prognosis. EDAR-associated death domain (EDARADD) regulates TRIM21 through PPARa and inhibits the ubiquitination and degradation of Snail1, a transcriptional repressor, thereby promoting colon cancer proliferation and epithelial-mesenchymal transition (EMT) (Yang et al., 2023). Compared to normal tissues, TRIM47 expression is significantly higher in colorectal cancer (CRC) tissues and is associated with poor prognosis. TRIM47 mediates the ubiquitination and degradation of mothers against decapentaplegic homolog 4 (SMAD4), leading to upregulation of C-C motif chemokine ligand 15 (CCL15) and C-C motif chemokine receptor 1 (CCR1) expression. The activation of the CCL15/CCR1 axis promotes the proliferation, invasion, and metastasis of CRC. Therefore, TRIM47 may be a potential target and prognostic marker for CRC therapy (Liang et al., 2019). Upregulation of TRIM65 is associated with poor prognosis. TRIM65 mediates the ubiquitination of ARHGAP35, leading to increased Rho GTPase activity and promoting the proliferation, invasion, migration, and metastasis of CRC to the liver and lungs. The TRIM65-ARHGAP35 axis can serve as a therapeutic target and prognostic marker in CRC (Chen et al., 2019). In contrast, TRIM16 is downregulated in CRC, and its expression level is linked to patient prognosis. TRIM16 interacts with Snail1 and ubiquitinates it, which inhibits EMT, invasion, migration, and metastasis in CRC (Ruan et al., 2021). Therefore, TRIM16 may serve as a marker for assessing disease progression and as a potential therapeutic target. Similarly, TRIM21 is downregulated in advanced human CRC and is associated with metastasis and poor prognosis. TRIM21 ubiquitinates mammalian STE20-like protein kinase 2 (MST2) through K63-linked chains, leading to inactivation of yes-associated protein (YAP) and reducing tumor invasion and metastasis (Liu et al., 2023). Collectively, TRIM proteins act as pivotal modulators of colorectal tumorigenesis and represent promising prognostic biomarkers and therapeutic targets (Figure 2).

#### 3.1.2 RNF family

The RNF family, a core subgroup of RING-type E3 ubiquitin ligases, includes multiple members that are frequently dysregulated in CRC, and their aberrant expression is closely associated with tumor progression, clinicopathological features, and patient prognosis by mediating the ubiquitination and degradation of key signaling molecules. The expression of RNF2 is increased in colon cancer tissues and is linked to various adverse clinicopathological features. RNF2 promotes the proliferation, migration, and invasion of SW480 cells by mediating the ubiquitination and degradation of interferon regulatory factor 4 (IRF4). Therefore, RNF2 could serve as a therapeutic and prognostic target for colorectal cancer (Wang et al., 2022). RNF6 is overexpressed in CRC patients and serves as an independent prognostic marker for poor outcomes. It promotes the ubiquitination and degradation of transducin-like enhancer protein 3 (TLE3), which results in the activation of the Wnt/β-catenin pathway. This activation promotes the proliferation, EMT, invasion, migration, and metastasis of colorectal cancer cells (Liu et al., 2018b). Additionally, RNF6 promotes the ubiquitination and degradation of Src homology 2 domain-containing protein tyrosine phosphatase 1 (SHP-1), which activates the JAK/STAT3 pathway and further promotes the proliferation, invasion, and metastasis of colorectal cancer cells (Liang et al., 2018). In summary, RNF6 acts as an oncogene and a potential biomarker in colorectal cancer development. RNF8 expression is increased in tumor tissues of CRC patients. It induces β-catenin ubiquitination, upregulates c-Myc expression, accelerates colon cancer proliferation and progression, and is linked to poor prognosis in patients (Ren et al., 2020). RNF126 promotes the proliferation and migration of CRC cells by ubiquitinating and degrading p53, which contributes to the development of drug resistance. Therefore, high RNF126 expression is also linked to an unfavorable prognosis (Wang S. et al., 2020). RNF148 is upregulated in most CRC tissues, and its expression is significantly associated with overall survival (OS) and clinicopathological parameters. RNF148 mediates the degradation of cation transport regulator-like protein 2 (CHAC2) via ubiquitination, promoting CRC cell proliferation and migration. Additionally, RNF148 induces acquired resistance by reducing CRC cells' sensitivity to 5-Fluorouracil (Liu S. et al., 2024). Collectively, different RNF family members (RNF2, RNF6, RNF8, RNF126, RNF148) exert oncogenic effects in CRC through distinct ubiquitination targets and signaling pathways, and their abnormal expression is closely linked to adverse clinicopathological outcomes and drug resistance, highlighting their critical roles in CRC pathogenesis and clinical significance.

#### 3.1.3 TRAF family

TRAF6, a member of the TRAF family of RING-type E3 ligases, exhibits dual oncogenic and tumor-suppressive roles in CRC progression, with its expression level closely linked to patient prognosis. Previous studies reveal the dual roles of TRAF6 in CRC progression. On one hand, Sphingosine kinase 1 (SPHK1) promotes colorectal cancer proliferation, invasion, and metastasis by regulating the autophagy process mediated by Unc-51 like kinase 1 (ULK1) ubiquitination induced by TRAF6 (Chen D. et al., 2024). Similarly, TRAF6 enhances the proliferation, invasion, migration, and lymphatic metastasis of CRC through the LPS-NF-κB-VEGF-C signaling pathway (Guangwei et al., 2022). Conversely, monocyte chemoattractant protein induced protein 1 (MCPIP1) inhibits the NF-κB signaling pathway in CRC by reducing the ubiquitination of TRAF6, thereby suppressing the proliferation and migration of CRC cells (Ye et al., 2023). On the other hand, TRAF6 inhibits CRC invasion and metastasis by regulating the selective autophagy-mediated degradation of β-catenin (Wu et al., 2019). TRAF6 exerts dual regulatory effects on CRC progression via different signaling pathways and molecular targets, providing valuable clinical insights for the treatment of CRC metastasis.

#### 3.1.4 SCF-type E3 ligases

Members of the FBX family, key components of SCF-type E3 ligases, exhibit distinct expression patterns and regulatory roles in CRC by mediating ubiquitination and degradation of target proteins. FBXL8 ubiquitinates and degrades the tumor suppressor gene TP53, promoting proliferation, invasion, migration, liver metastasis, and stem-like properties in CRC. FBXL8 is considered as a potential target and a poor prognostic marker in CRC (Yao et al., 2023). Similarly, FBXW10 promotes colorectal cancer proliferation, migration, angiogenesis, and liver metastasis by mediating the ubiquitination and degradation of large tumor suppressor 2 (LATS2). Therefore, the FBXW10-LATS2 axis could be a potential target for therapy and a prognostic marker in CRC (Zhang Z. Y. et al., 2023). In summary, FBXL8 and FBXW10 are upregulated in CRC, and patients with high expression levels have a poor prognosis. In contrast, FBX8 inhibits CRC proliferation, invasion, and metastasis by ubiquitinating and degrading glutathione S-transferase pi-1 (GSTP1) (FeiFei et al., 2019).

### 3.2 Liver cancer

#### 3.2.1 TRIM family

**FIGURE 3:**
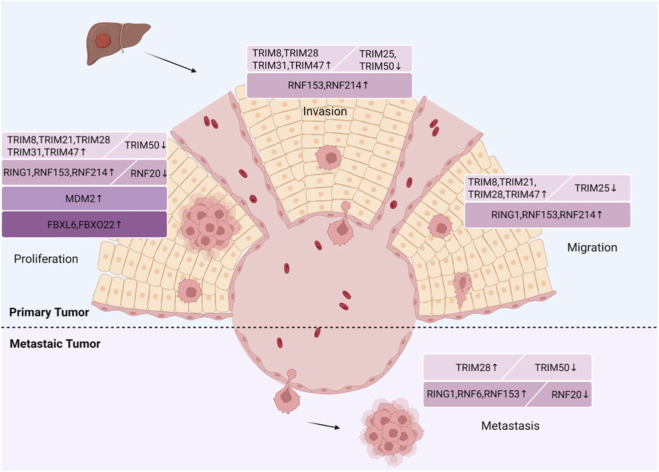
From an anatomical perspective of the liver, RING-type E3 ligases have either promoting or inhibitory effects at all stages of liver cancer development.

TRIM family members, a subgroup of RING-type E3 ligases, show aberrant expression in hepatocellular carcinoma (HCC) and exert dual oncogenic or tumor-suppressive roles by mediating target protein ubiquitination. The expression of TRIM8 is increased in *HCC* tissues and is linked to aggressive behavior and poor prognosis in HCC patients. TRIM8 ubiquitinates and degrades hepatocyte nuclear factor 1α (HNF1α), which promotes the proliferation, colony formation, invasion, and migration of HCC cells. Therefore, targeting the TRIM8-HNF1α axis could be a promising therapeutic strategy for HCC (Peng et al., 2024). Elevated TRIM21 expression is also linked to HCC progression and worse survival rates. TRIM21 inhibits the activity of mammalian STE20-like protein kinase 1 (MST1), which affects the phosphorylation of YAP and promotes the proliferation and migration of liver cancer cells (Shu et al., 2024). Additionally, TRIM21 has been identified as a key regulator of sorafenib resistance in HCC. The expression levels of UBE2S and TRIM28 are elevated in HCC tissues, and the prognosis for HCC patients is poor. UBE2S, as a crucial enzyme in the ubiquitination pathway, interacts with TRIM28 in the nucleus, enhancing the ubiquitination and degradation of p27. This process promotes the proliferation, invasion, migration, and metastasis of HCC cells. Targeting the UBE2S–TRIM28–p27 axis may serve as a promising new approach for treating HCC (Zhang et al., 2021). TRIM31 expression is increased in HCC tissues and is linked to disease progression. It promotes HCC development by targeting and degrading the tuberous sclerosis complex 1 (TSC1)-TSC2 complex, thereby activating the mTORC1 signaling pathway (Guo et al., 2018). TRIM47 is highly expressed in HCC tissues, and its levels are associated with clinical stage and patient prognosis. TRIM47 can ubiquitinate cysteine dioxygenase type 1 (CDO1), leading to the downregulation of CDO1 expression, which inhibits ferroptotic cell death in HCC and promotes proliferation, invasion, and migration (Zhang J. et al., 2024). However, TRIM25 ubiquitinates and degrades metastasis-associated protein 1 (MTA1). By inhibiting MTA1, TRIM25 suppresses the migration and invasion of HCC cells and slows disease progression, indicating its potential as a diagnostic biomarker (Zang et al., 2017). Similarly, TRIM50 expression is reduced in HCC tissues and inversely linked to disease progression. TRIM50 ubiquitinates SNAIL, suppresses tumor proliferation, colony formation, and effectively blocks the EMT process in tumor cells. This reduces the invasion and metastasis capability of tumor cells (Ma et al., 2018). Different TRIM members regulate HCC progression via distinct molecular axes, correlating with patient prognosis and providing potential therapeutic strategies for HCC (Figure 3).

#### 3.2.2 RNF family

Distinct RNF subtypes participate in HCC pathogenesis through targeting different tumor-related substrates, whereas individual members exhibit inconsistent expression and biological functions. RING1 is upregulated in HCC patients and linked to poor clinical outcomes. RING1 can target p53 and mediate its ubiquitination and degradation, promoting the proliferation, migration, and metastasis of HCC cells (Shen et al., 2018). As a result, RING1 serves as a potential biomarker and therapeutic target for cancer prognosis and treatment. Similarly, high expression of RNF6 is strongly associated with malignant clinical features and poor survival in HCC patients. RNF6 binds to and ubiquitinates forkhead box protein A1 (FoxA1), which promotes metastasis, EMT, and increases radioresistance in HCC (Cai et al., 2019). Membrane-associated RING-CH5, also known as RNF153, is significantly overexpressed in HCC cells and is associated with poor patient outcomes. RNF153 promotes the proliferation, invasion, migration, and metastasis of HCC cells by inducing the ubiquitination and degradation of p53, thereby accelerating the progression of HCC (Cai X. et al., 2024). RNF214 is also highly expressed in HCC and correlates with poor prognosis. It mediates the ubiquitination of the transcriptional enhancer factor domain (TEAD), enhances the binding to YAP, and activates Hippo signaling. This process promotes proliferation, invasion, and migration while suppressing apoptosis, indicating RNF214 as a potential target for HCC therapy (Lin et al., 2024). Conversely, the mRNA expression level of RNF20 in the serum of HCC patients is decreased. Patients with higher RNF20 expression have a better postoperative survival rate compared to those with lower levels. RNF20 enhances the ubiquitination of NOD-like receptor protein 3 (NLRP3), which inhibits the proliferation and metastasis of liver cancer cells (Liu D. et al., 2024). The RNF20-NLRP3 signaling axis inhibits liver cancer progression and can serve as a biomarker for assessing postoperative survival rates. Notably, RING1, RNF6, RNF153 and RNF214 facilitate malignant progression of HCC via ubiquitin modification, while RNF20 restrains tumor growth, revealing their opposing regulatory patterns in hepatocellular carcinoma.

#### 3.2.3 MDM family

Beyond canonical RING-type E3 members, auxiliary regulatory proteins govern HCC progression by facilitating the MDM2-dependent ubiquitination and degradation of p53. Myosin light chain 6B (MYL6B) is a myosin light chain that promotes the binding of MDM2 to p53, leading to p53 ubiquitination and degradation. This process enhances the proliferation of HCC cells and suppresses cell apoptosis (Xie et al., 2018). Similarly, heat shock protein gp96 mediates the ubiquitination and degradation of p53 and promotes the proliferation of liver cancer cells by interacting with MDM2 (Wu B. et al., 2015). MYL6B and gp96 both accelerate HCC cell proliferation and inhibit apoptosis by strengthening the MDM2-p53 interaction and subsequent p53 ubiquitin depletion.

#### 3.2.4 SCF-type E3 ligase

Several F-box family members exhibit aberrant upregulation in HCC and modulate the stability of downstream substrates to drive tumor progression through distinct ubiquitin-dependent mechanisms. FBXL6 is significantly upregulated in HCC tissues. It inhibits the ubiquitination and degradation of heat shock protein 90 alpha A1 (HSP90AA1), leading to the stabilization of HSP90AA1 and activation of the c-MYC signaling pathway. These effects support HCC proliferation and progression (Shi et al., 2020). Targeting FBXL6 could help slow the progression of HCC. Additionally, FBXO22 is highly expressed in HCC tissues and is associated with poor patient outcomes. Through its F-box domain, FBXO22 ubiquitinates p21, which promotes the proliferation of HCC cells (Zhang L. et al., 2019). Furthermore, FBXO22 promotes the ubiquitination and degradation of Krüppel-like factor 4 (KLF4), reducing its inhibitory effect on downstream tumor suppressor genes. This process further promotes hepatocellular carcinoma cell proliferation and disease progression (Tian et al., 2015). Mechanistically, FBXL6 stabilizes HSP90AA1 to activate c-MYC signaling, while FBXO22 facilitates the ubiquitination degradation of p21 and KLF4, jointly accelerating the malignant proliferation and progression of HCC.

### 3.3 Pancreatic cancer

#### 3.3.1 TRIM family

**FIGURE 4:**
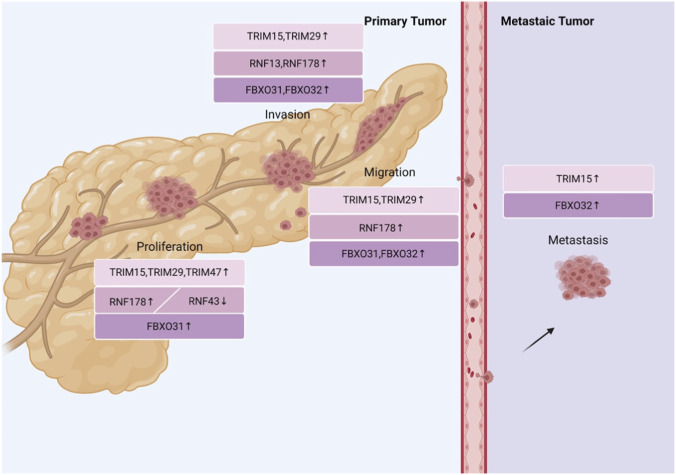
Currently, there is no better targeted treatment for pancreatic cancer. The figure summarizes the factors related to pancreatic cancer progression based on RING-type E3 ligases, offering theoretical support for selecting treatment targets for pancreatic cancer.

Multiple TRIM family members are abnormally overexpressed in pancreatic cancer and correlate with malignant clinicopathological features, regulating lipid metabolism and glycolysis to facilitate tumor progression via distinct ubiquitination-dependent mechanisms. The expression of TRIM15 in pancreatic cancer tissues is significantly higher than in adjacent normal tissues and is closely associated with clinicopathological features and poor prognosis in patients. TRIM15 influences lipid metabolism via the apolipoprotein A1 (APOA1)-low-density lipoprotein receptor (LDLR) axis and promotes the invasion, migration, and metastasis of pancreatic cancer (Sun et al., 2021). Similarly, TRIM15 can ubiquitinate insulin-like growth factor 2 mRNA binding protein 2 (IGF2BP2) and improve the function and stability of Toll-like Receptor 4 (TLR4), thereby promoting colony formation, proliferation, invasion, and migration of pancreatic cancer cells (Cai H. et al., 2024). Nucleosome assembly protein 1-like 5 (NAP1L5) can recruit TRIM29, mediate the ubiquitination of PH domain leucine-rich repeat protein phosphatase 1 (PHLPP1), and activate the AKT/mTOR signaling pathway. This process promotes the proliferation, invasion, and migration of pancreatic ductal adenocarcinoma (Xiao et al., 2023). TRIM47 is also significantly upregulated in pancreatic cancer tissues, indicating a poor prognosis for patients. TRIM47 ubiquitinates and degrades fructose-1,6-biphosphatase (FBP1), which enhances aerobic glycolysis and promotes the proliferation of pancreatic cancer cells (Li et al., 2021). In conclusion, TRIM15 modulates lipid metabolism and innate immune signaling, NAP1L5-recruited TRIM29 activates the AKT/mTOR cascade, and TRIM47 enhances aerobic glycolysis; these molecules cooperatively drive pancreatic tumor malignancy and represent promising therapeutic targets for combined antitumor strategies (Figure 4).

#### 3.3.2 RNF family

Different RNF family members exert distinct regulatory patterns in pancreatic cancer, whose expression and downstream signaling axes are modulated through specific upstream molecules and ubiquitin modification. Research shows that Tenascin-C can increase the expression of RNF13 in pancreatic ductal adenocarcinoma, and higher levels of RNF13 are significantly associated with the histological grade of pancreatic cancer. RNF13 enhances the invasive ability of pancreatic cancer cells and promotes cancer progression by increasing matrix metalloproteinase-9 (MMP-9) activity (Zhang et al., 2009). Membrane-associated Ring-CH-type finger 8 (MARCH8), also known as RNF178, interacts with protein tyrosine phosphatase non-receptor type 4 (PTPN4), mediating its ubiquitination and degradation. This process activates the signal transducer and activator of transcription 3 (STAT3) signaling pathway. It promotes the proliferation, invasion, and migration of pancreatic cancer cells and inhibits cell apoptosis (Chen et al., 2023). However, RNF43 has been shown to inhibit pancreatic cancer cell proliferation by suppressing the BRAF/MEK signaling pathway (Hsu et al., 2024). RNF13 and MARCH8 facilitate pancreatic tumor progression through the MMP-9 and STAT3 pathways respectively, whereas RNF43 restrains malignant growth by inhibiting BRAF/MEK signaling, highlighting their opposite roles and providing valuable clues for targeted drug research.

#### 3.3.3 SCF-type E3 ligases

Several F-box proteins are abnormally upregulated in pancreatic cancer and modulated by epigenetic modification, participating in cell cycle regulation and malignant progression through substrate ubiquitination. FBXO31 plays important roles in cell cycle regulation and DNA damage repair. It is highly expressed in pancreatic cancer and associated with poor prognosis. Methyltransferase-like 3 (METTL3) enhances FBXO31 expression through m^6^A modification. In pancreatic cancer cells, overexpression of FBXO31 can cause ubiquitination and degradation of Sirtuin 2 (SIRT2), thereby promoting the proliferation, invasion, and migration of these cells (Chen K. et al., 2024). Another study has shown that FBXO32 is highly expressed in pancreatic cancer and associated with poor prognosis. FBXO32 can ubiquitinate and enhance the activity of eukaryotic translation elongation factor 1 alpha 1 (eEF1A1), promoting the invasion, migration, and metastasis of pancreatic ductal adenocarcinoma (Su et al., 2024). In summary, METTL3-mediated m^6^A modification elevates FBXO31 to degrade SIRT2 and facilitate tumor growth, while FBXO32 activates eEF1A1, both driving the metastatic progression of pancreatic cancer.

### 3.4 Gastric cancer

#### 3.4.1 TRIM family

**FIGURE 5:**
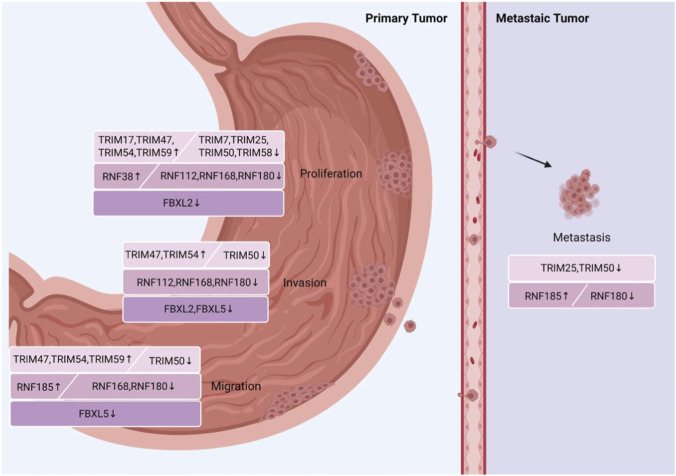
Treatment options for metastatic gastric cancer are limited. RING-type E3 ligases have already played a preliminary regulatory role in the early stage of gastric cancer metastasis.

As key regulators in gastric cancer (GC) progression, TRIM family members exhibit distinct expression patterns—most are upregulated and promote tumorigenesis, while a few are downregulated and exert tumor-suppressive effects, participating in GC development through diverse molecular mechanisms. Researchers have found that TRIM17 is upregulated in *GC*compared to normal tissues, and patients with this disease often face a poor prognosis. TRIM17 promotes the survival and progression of GC cells by regulating the stability of the pro-apoptotic protein Bcl-2-associated X protein (BAX), thereby inhibiting cell apoptosis (Shen et al., 2023). Similarly, TRIM47 is significantly upregulated in GC tissues and linked to poor patient prognosis. It promotes proliferation, invasion, and migration through the CYLD-NF-κB pathway (Wang et al., 2024). TRIM54 is also upregulated in GC tissues, with higher expression levels indicating a worse prognosis. It promotes proliferation, invasion, and migration by ubiquitinating and degrading Filamin C (Cao et al., 2022). TRIM59 is highly expressed in GC, and patients’ survival time is significantly reduced. TRIM59 promotes tumor growth and spread by facilitating the ubiquitination and degradation of p53 (Zhou et al., 2014). In contrast, TRIM7 expression is reduced in GC tissues, and patients with low TRIM7 levels have a poor prognosis. TRIM7 ubiquitinates and degrades solute carrier family 7 member 11 (SLC7A11), inhibits the SLC7A11-glutathione peroxidase 4 (GPX4) axis, and induces ferroptosis in GC cells while also suppressing their growth (Chen Q. et al., 2024). The study also shows that decreased expression of TRIM25 and increased expression of the transcription factor SP1 in GC tissues are positively associated with poor prognosis. The anti-GC peptide JP3, which targets matrix metalloprotease-2 (MMP2), stabilizes TRIM25, aids in the degradation of SP1, reduces MMP2 expression, and ultimately inhibits angiogenesis, proliferation, and metastasis in GC (Chen et al., 2020). TRIM50 is downregulated in gastric cancer tissues, which is associated with malignant behavior and a poor prognosis of GC. TRIM50 inhibits c-MYC transcription and suppresses proliferation, migration, and metastasis of GC by regulating the ubiquitination, degradation, and nuclear translocation of the transcription factor JUP (Hu et al., 2023). Additionally, TRIM50 can degrade phosphoglycerate kinase 1 (PGK1) via ubiquitination, while also inhibiting macrophage M2 polarization and reducing the glycolytic activity, proliferation, invasion, and migration of GC cells (Gu et al., 2024). TRIM58 expression is notably reduced in GC tissues. As a tumor-suppressive E3 ubiquitin ligase, it ubiquitinates and degrades β-catenin, thereby preventing the proliferation of GC cells (Liu et al., 2020). In conclusion, TRIM17, TRIM47, and TRIM54 are upregulated in GC and drive tumor progression via distinct pathways, while TRIM7, TRIM25, TRIM50, and TRIM58 are downregulated and exert tumor-suppressive effects, providing valuable targets for GC diagnosis, prognosis, and targeted therapy (Figure 5).

#### 3.4.2 RNF family

Members of the RNF family exhibit distinct expression patterns in gastric cancer (GC), with some being upregulated and others downregulated, and their mediated ubiquitination of specific substrates regulates GC progression, closely correlating with patient OS and prognosis. RNF38 is highly expressed in GC tissues and is associated with poor prognosis. It mediates the ubiquitination and degradation of SHP-1 and promotes STAT3 signaling, which can lead to the proliferation of GC cells (Zhang et al., 2018). High expression of RNF185 in tumor tissues of GC patients is significantly linked to shorter OS. RNF185 promotes the migration and metastasis of GC cells by mediating the degradation of JWA (also known as ARL6IP5) through the ubiquitin-proteasome pathway. Inhibiting RNF185 could be a new strategy for treating metastatic GC patients (Qiu et al., 2018). Forkhead Box M1 (FOXM1) is highly expressed in patients with advanced GC, while RNF112 expression is decreased. RNF112 suppresses the proliferation and invasion of GC cells by mediating the ubiquitination and degradation of FOXM1. The above study suggests that the RNF112-FOXM1 axis could be a potential target for GC treatment (Zhang S. et al., 2023). Another study shows that RNF168 is downregulated in human GC tissues, and its low expression is linked to shorter OS. RNF168 interacts with Ras homolog gene family member C (RHOC) and promotes its degradation by mediating its ubiquitination. The degradation of RHOC can decrease the levels of histone deacetylase 1 (HDAC1), which helps inhibit proliferation, invasion, and migration in gastric cancer (Xu et al., 2021). Protocadherin 10 (PCDH10) is downregulated in GC and positively associated with RNF180 expression in human GC tissues. Low expression of both genes is linked to poor prognosis and malignant clinical features. Mechanism studies have shown that RNF180 increases the expression of PCDH10 by promoting ubiquitin-dependent degradation of DNA methyltransferase 1 (DNMT1), thereby inhibiting the proliferation, invasion, migration, and metastasis of GC cells. Therefore, the RNF180/DNMT1/PCDH10 axis could be a potential target for GC therapy (Zhang N. et al., 2023). Collectively, RNF38 and RNF185 are upregulated in GC and drive tumor proliferation, migration, and metastasis via the SHP-1/STAT3 and JWA pathways, respectively, while RNF112, RNF168, and RNF180 are downregulated and exert tumor-suppressive effects through distinct molecular axes, highlighting their potential as diagnostic and therapeutic targets for GC.

#### 3.4.3 SCF-type E3 ligases

Members of the FBX family exhibit distinct expression patterns in GC and function as substrate recognition subunits of the SCF ubiquitin ligase complex, regulating GC progression by mediating ubiquitination and proteasomal degradation of specific target proteins. FBXL2 is downregulated in GC tissues. It acts as a substrate recognition subunit of the SCF ubiquitin ligase complex, mediating ubiquitination and proteasomal degradation of FoxM1, thereby reducing the proliferation and invasion abilities of GC cells (Li et al., 2016). In contrast, the expression levels of FBXL5 and Snail1 are negatively correlated in GC tissues. Patients with low FBXL5 expression and high Snail1 expression are more likely to face a higher risk of metastasis and a worse prognosis. FBXL5 ubiquitinates and degrades Snail1, thereby inhibiting the invasion and migration of GC cells. This highlights the key role of the FBXL5-Snail1 axis in GC progression (Wu W. et al., 2015). Collectively, FBXL2 is downregulated in GC and exerts a tumor-suppressive effect by targeting FoxM1 for ubiquitination and degradation to inhibit GC cell proliferation and invasion, while FBXL5 negatively regulates Snail1 expression via ubiquitination, and the FBXL5-Snail1 axis plays a crucial role in regulating GC metastasis and patient prognosis.

### 3.5 Esophageal cancer

#### 3.5.1 TRIM family

**FIGURE 6:**
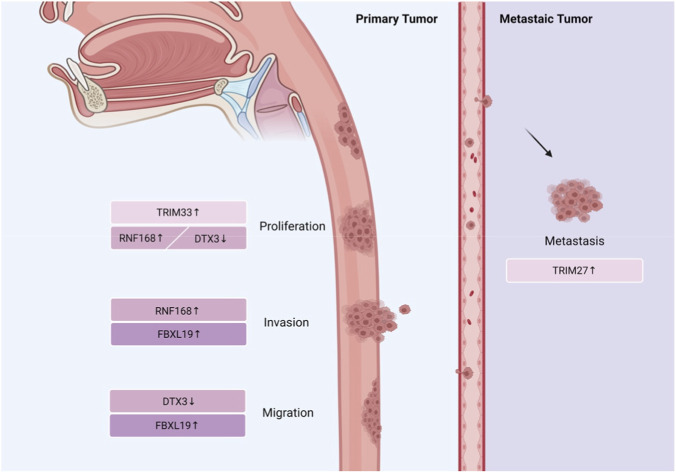
TRIM, RNF, and SCF-type E3 ligases families play different roles in regulating the occurrence and progression of esophageal cancer. In response to individual differences in RING-type E3 ligases expression, clinicians can select more appropriate therapeutic targets for esophageal cancer patients in future targeted therapies.

Esophageal squamous cell carcinoma (ESCC) is a highly malignant digestive tract tumor characterized by chemotherapy resistance and the lack of specific diagnostic and therapeutic biomarkers, while TRIM family members (TRIM27 and TRIM33) play crucial regulatory roles in ESCC progression through ubiquitination-dependent mechanisms. The study indicates that TRIM27 regulates the expression of L1 cell adhesion molecule (L1CAM) by ubiquitinating Krüppel-like factor 12 (KLF12), leading to cisplatin resistance and tumor metastasis (Zhang H. et al., 2024). Additionally, TRIM33 is highly expressed in ESCC tissues and cell lines and is linked to poor clinical outcomes. TRIM33 regulates the ubiquitination of p53, reduces its stability, promotes aerobic glycolysis, and encourages the proliferation of ESCC cells (Xia et al., 2024). In summary, TRIM27 mediates the ubiquitination of KLF12 to regulate L1CAM expression, thereby promoting cisplatin resistance and tumor metastasis in ESCC, while TRIM33 modulates p53 stability and aerobic glycolysis to drive ESCC cell proliferation. Both TRIM27 and TRIM33 are closely associated with ESCC malignant progression and can serve as potential biomarkers for evaluating the risk of ESCC progression (Figure 6).

#### 3.5.2 RNF family

Different RNF family members and their homologous E3 ligases display abnormal expression patterns in esophageal cancer, and their mediated ubiquitination of specific substrates modulates tumor progression, which is closely correlated with patient survival and therapeutic potential. RNF168 exhibits a high rate of gene amplification in esophageal cancer, and its amplification is linked to decreased survival. It promotes the proliferation and invasion of esophageal cancer cells by increasing the stability of signal transducer and activator of transcription 1 (STAT1) and activating the JAK-STAT signaling pathway (Yu et al., 2019). In contrast, Deltex E3 ubiquitin ligase 3 (DTX3, also known as RNF154) is downregulated in human esophageal cancer samples, while Notch receptor 2 (NOTCH2) is highly expressed. DTX3 inhibits the proliferation and migration of esophageal cancer cells by mediating the ubiquitination and degradation of NOTCH2, thereby suppressing NOTCH signaling activity. This suggests that DTX3 may be a potential target for esophageal cancer treatment (Ding et al., 2020). Overall, RNF168 is frequently amplified in esophageal cancer and facilitates tumor cell proliferation and invasion by enhancing STAT1 stability and activating the JAK-STAT signaling pathway, whereas DTX3 (also referred to as RNF154) is downregulated and exerts a tumor-suppressive effect by mediating NOTCH2 ubiquitination and degradation to inhibit NOTCH signaling, indicating its potential as a therapeutic target for esophageal cancer.

#### 3.5.3 SCF-type E3 ligases

Given the high malignancy of esophageal cancer and the lack of specific diagnostic and therapeutic targets, FBXL19, as a key regulatory factor, participates in the progression of esophageal cancer through ubiquitination-dependent mechanisms, which is closely related to tumor invasion and chemotherapy response. FBXL19 mediates the ubiquitination and degradation of Rac3. This process enhances the TGFβ1-induced downregulation of E-cadherin in esophageal cancer cells. The above mechanism promotes the EMT process, which benefits tumor cells by increasing their invasion and migration capabilities (Dong et al., 2014). To summarize, FBXL19 mediates Rac3 ubiquitination, enhances TGFβ1-induced E-cadherin downregulation, and promotes esophageal cancer cell invasion and EMT, serving as a key factor in tumor progression.

## 4 The connection between RING-type E3 ligases and drug resistance

**FIGURE 7:**
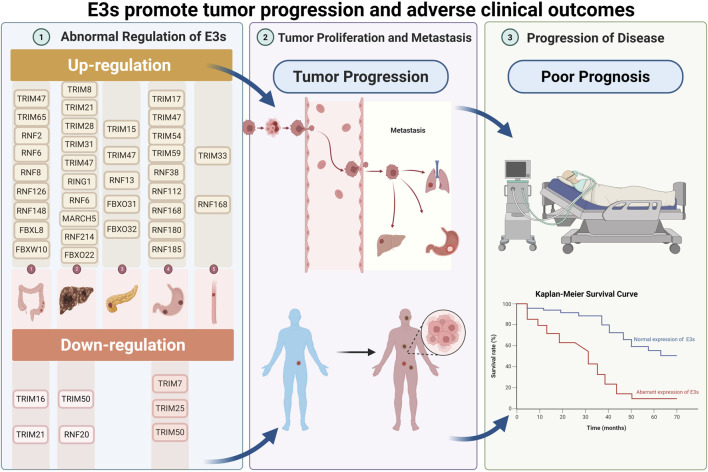
Each enzyme in the figure is either upregulated or downregulated, and all are related to tumor progression and poor prognosis, including tumor growth and metastasis, reduced OS, resistance to anticancer drugs, and deteriorating patient outcomes.

In digestive system tumors, acquired resistance after chemotherapy poses a significant challenge that results in treatment failure. Changes in the expression levels of RING-Type E3 Ligases are also closely associated with poor patient prognosis (Figure 7). A comprehensive understanding of drug resistance mechanisms is essential for developing effective strategies to overcome it (Nobili et al., 2015). RING-type E3 ligases, as crucial enzymes in the ubiquitination pathway, regulate various biological processes such as cell proliferation, apoptosis, and drug resistance by mediating the ubiquitination of target proteins (Kleiger and Mayor, 2014; Swatek and Komander, 2016; Uchida and Kitagawa, 2016). These enzymes can influence how tumor cells respond to chemotherapy drugs, targeted therapies, and immunotherapy by interacting with drug targets, activating drug resistance signaling pathways, or changing the tumor microenvironment (Satija et al., 2013; Liu et al., 2018a; Wang et al., 2017). Therefore, detailed research on the specific mechanism of RING-type E3 ligase in drug resistance of digestive system tumors provides a direction for developing new intervention strategies targeting this pathway and has significant basic research and clinical translational value.

Conventional treatment strategies for CRC include chemotherapy [5-Fluorouracil (5-FU), Irinotecan, Oxaliplatin, etc.,], targeted therapy (Bevacizumab, Cetuximab, Fruquintinib, Regorafenib, etc.), and immunotherapy (Abedizadeh et al., 2024). Currently, evidence shows that ubiquitination plays a key role in mediating drug resistance and metastatic potential in CRC by regulating various signaling pathways. For example, TRIM21, identified as a target of extracellular signal-regulated kinase 2 (ERK2) in KRAS-mutant CRC, contributes to resistance to regorafenib treatment (Ye et al., 2024). TRIM25 inhibits the interaction between TRAF6 and enhancer of zeste homolog 2 (EZH2), thereby blocking TRAF6-mediated ubiquitination and degradation of EZH2. It helps maintain cancer stem cell characteristics and increases oxaliplatin resistance in CRC cells (Zhou et al., 2021). TRIM47 is overexpressed in CRC tissues and linked to resistance to 5-FU (Liang et al., 2019). RNF126 promotes resistance to 5-FU by increasing the ubiquitination and breakdown of p53 (Wang S. et al., 2020). Cyclophilin B interacts with MDM2 to promote ubiquitination and degradation of wild-type p53, thereby inhibiting oxaliplatin-induced apoptosis and promoting drug resistance (Choi et al., 2018). Furthermore, CRC stem cells develop irinotecan resistance through FBXW7-mediated degradation of c-Myc (Izumi et al., 2017). Related studies have shown that BARD1 is a RING-type E3 ligase. It confers resistance to oxaliplatin in colorectal cancer cells by interacting with metallothionein 2A (MT2A) and BRCA1 (Zhao et al., 2020).

Growing evidence indicates that RING-type E3 ligases play a crucial role in acquired drug resistance in HCC. TRIM21 enhances the proliferation and sorafenib resistance of HCC through the TRIM21-MST1-YAP pathway (Shu et al., 2024). While Nogo-B receptor (NgBR), a type I single-pass transmembrane receptor, mediates ubiquitination and degradation of the p53 protein by activating the PI3K-Akt-MDM2 pathway, it thereby increases liver cancer cells’ resistance to 5-FU (Dong et al., 2016). Transforming growth factor-β-activated kinase 1 (TAK1) is ubiquitinated by the E3 ubiquitin ligase FBXW2. However, metadherin can bind to FBXW2 mRNA and accelerate its degradation, leading to decreased FBXW2 expression and increased TAK1 protein levels. TAK1 activates various downstream signaling pathways to promote the proliferation and survival of liver cancer cells. At the same time, TAK1 can also make liver cancer cells resistant to sorafenib (Xia et al., 2021). Furthermore, in HCC, the tumor suppressor FBW7 ubiquitinates chromodomain helicase DNA-binding protein 3 (CHD3), thereby inhibiting metastasis, stemness, and resistance to oxaliplatin in HCC cells (Li et al., 2024).

RING-type E3 ligases also play a role in drug resistance in other digestive system tumors. For example, RNF138 is highly expressed in cisplatin-resistant GC cell lines, and its expression increases significantly after cisplatin withdrawal in GC patients (Lu et al., 2018). FBXL5, a member of the SCF complex family, increases the sensitivity of the GC cell line MKN-28 to cisplatin (Wu et al., 2016). Similarly, FBW7 functions as a tumor suppressor and mediates the ubiquitination and degradation of various oncoproteins. In GC cells, microRNA expression is inhibited after targeting FBW7, which prevents it from effectively performing its tumor suppressor function, leading to increased proliferation of GC cells and resistance to chemotherapy drugs such as docetaxel, cisplatin, 5-fluorouracil, and targeted drugs like trastuzumab (Gao et al., 2020; Lin et al., 2020; Zhang et al., 2016; Eto et al., 2015). In esophageal cancer, TRIM37 increases resistance to cisplatin via the NF-κB signaling pathway (Wang H. et al., 2021). Moreover, MDM2 plays a crucial role in drug resistance in pancreatic cancer. TP53 mutations are highly prevalent in pancreatic cancer, and MDM2 is a major negative regulator of p53 (Wang et al., 2014; Wang W. et al., 2020). Therefore, developing small-molecule inhibitors that target MDM2 could be a promising approach to overcoming acquired drug resistance in pancreatic cancer patients.

## 5 Therapeutic strategies targeting RING-Type E3 ligases

### 5.1 Small molecule inhibitors targeting E3 ligases

**FIGURE 8:**
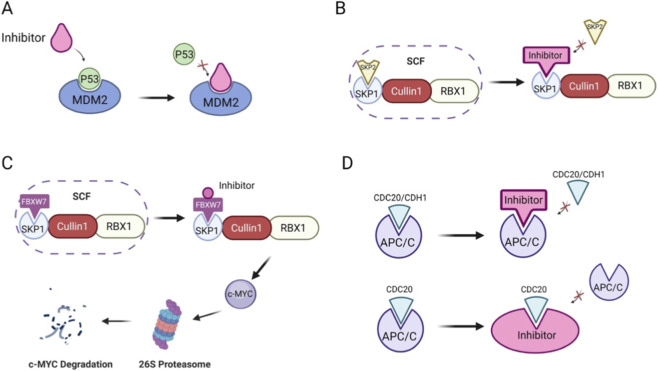
Therapeutic mechanisms of small-molecule inhibitors targeting RING-type E3 ligases in digestive system tumors. MDM2 inhibitors can bind to the p53 binding site of MDM2, blocking the interaction between MDM2 and p53 **(A)**. Inhibitors of SKP2 can competitively bind to the binding sites of SKP1 and SKP2 in the SCF complex exert an anti-tumor effect **(B)**. Another inhibitor of the SCF complex also has anti-tumor effects by promoting ubiquitination and degradation of the c-MYC signaling pathway **(C)**. CDC20 and CDH1 activate APC/C and are involved in cell mitosis. APC/C inhibitors prevent this activation process, suppress excessive mitosis in tumor cells, and regulate the cell cycle and apoptosis of tumor cells **(D)**.

We have summarized in detail that RING-type E3 ligases cause dysfunction of related signaling pathways by regulating protein expression, which further influences the progression of digestive system tumors. Therefore, the development of compounds targeting E3 ligases has attracted increasing attention. Currently, small molecule inhibitors for solid tumors mainly focus on targets such as MDM2, SCF, IAP, and APC/C (Figure 8). At present, the small molecule inhibitors used in clinical research primarily target MDM2 (Table 1). Other inhibitors are still in the research and development stage and will take some time before they are applied to patients.

**TABLE 1:** Clinical studies related to MDM2 inhibitors.

Small compound	Tumor types	Phase	NCT number
Milademetan	Advanced solid tumors, lymphoma	Phase I/II/III	NCT01877382 NCT05012397 NCT04979442
RG7388 (Idasanutlin)	Solid tumors, AML, ALL, stage III breast cancer	Phase I/II/III	NCT02545283 NCT04029688 NCT03566485 NCT03850535
APG-115	Advanced solid tumors, lymphoma, liposarcoma, AML, CMML, MDS, melanoma	Phase I/II	NCT04358393 NCT04496349 NCT04785196 NCT03611868
SAR405838	Advanced solid tumors	Phase I	NCT01636479
AMG-232	Advanced solid tumors, multiple myeloma, AML, melanoma	Phase I	NCT01723020 NCT03041688 NCT03031730 NCT02110355
CGM097	Solid tumor with p53 wild type status	Phase I	NCT01760525
HDM201	AML, advanced soft-tissue sarcoma, liposarcoma, advanced solid and hematological TP53wt tumors, malignant solid tumor	Phase I/II	NCT04496999 NCT05180695 NCT03714958 NCT02343172 NCT02143635 NCT04116541
BI 907828	Solid tumors, liposarcoma	Phase I/II/III	NCT05512377 NCT05218499 NCT06058793 NCT06619509 NCT03449381
RG7112	Advanced solid tumors, hematologic neoplasms	Phase I	NCT00559533 NCT00623870
MK-8242	Solid tumors, AML	Phase I	NCT01463696 NCT01451437

#### 5.1.1 MDM2

As a tumor suppressor protein, p53 plays a crucial role in regulating the cell cycle, maintaining genome stability, inducing cell senescence, and other related processes. Mutations in the TP53 gene are often linked to the development of cancer (Levine, 1997). MDM2 serves as a negative regulator of p53, and an imbalance in the MDM2-p53 axis can lead to tumor progression (Clark et al., 2022). Small-molecule inhibitors that target MDM2 block its interaction with p53, leading to p53 reactivation. Nutlin, an imidazoline analogue, binds competitively to the p53 binding site on MDM2, preventing MDM2–p53 interaction and inhibiting tumor cell proliferation (Vassilev et al., 2004). Nutlin has been studied in various hematological and solid tumors. However, its use is limited because it is only effective in tumors with wild-type p53, showing no effect in p53-mutated or p53-deficient tumors (Sarisozen et al., 2019). Therefore, several derivatives targeting Nutlin, such as RG112 and RG7388, have been shown to have higher drug activity, but their anti-tumor functions need further confirmation (Tisato et al., 2017). AMG232, a piperidinone inhibitor, selectively binds to the MDM2 protein and restores p53 transcriptional activity. AMG232 has shown preliminary results in clinical studies and has demonstrated good safety and tolerability (Fang et al., 2020). Other compounds targeting MDM2, such as RITA, APG-115, PRIMA, Serdemetan, HLI373, and HLI98, also exert anti-tumor effects by inhibiting the MDM2-p53 interaction (Issaeva et al., 2004; Peuget et al., 2024; Herman et al., 2011; Kitagaki et al., 2008; Yang et al., 2005; Bykov et al., 2002; Chargari et al., 2011).

#### 5.1.2 SKP2

SKP2 is a substrate recognition subunit of the SCF ubiquitin ligase complex. It regulates the cell cycle and promotes tumor cell proliferation by ubiquitinating and degrading P27. Inhibitors targeting SKP2, such as the C series compounds (C1, C2, C16, C20), induce cell cycle arrest and lead to cell death by preventing SKP2 from binding to P27 (Wu et al., 2012). Similarly, compound A prevents SKP2 from binding to the SCF complex, also resulting in cell death (Chen et al., 2008). Additionally, compound #25 (C25), as a SKP2-targeting agent, exhibits anti-tumor activity by blocking the interaction between SKP2 and SKP1 (Chan et al., 2013).

#### 5.1.3 SCF complex

FBXW7 is a core component of the SCF ubiquitin ligase complex, which is an essential tumor suppressor protein and plays a central role in the cell cycle, apoptosis, stem cell maintenance, and tumor development (Yeh et al., 2018). Oridonin, a natural tetracyclic diterpenoid, activates FBXW7, which results in the ubiquitination and proteasome-dependent degradation of c-MYC (Huang et al., 2012). Additional study has shown that SCF-I2, a biplanar dicarboxylic acid compound, has potential antitumor activity by inhibiting substrate binding and ubiquitination of SCFCdc4 (Orlicky et al., 2010).

#### 5.1.4 APC/C

APC/C plays a central role in ensuring the accuracy of cell division by mediating the ubiquitination and degradation of specific substrates. It is a key regulator of the cell cycle in eukaryotic cells. The activity of APC/C depends on its association with co-activators, primarily cell division cycle protein 20 (CDC20) and CDC20 homolog 1 (CDH1). N-4-Tosyl-L-arginine methyl ester (TAME) binds to APC and prevents its activation by CDC20 and CDH1 (Zeng et al., 2010). Apcin binds to CDC20, directly inhibits the catalytic activity of APC/C, and blocks mitotic exit (Sackton et al., 2014).

### 5.2 Current status and potential of PROTAC-Based therapeutic strategies

Unlike conventional inhibitors that only inhibit protein activity, the proteolysis targeting chimera (PROTAC) are bifunctional small molecule compounds that use the UPS to induce selective degradation of intracellular proteins (Khan et al., 2020). In contrast to small-molecule inhibitors, PROTACs completely eliminate the target protein, thereby reducing the dose dependence required to achieve a therapeutic effect, thereby blocking its function and reducing the probability of development of resistance.

Each PROTAC is composed of three components: one that binds to the target protein and the other that recruits the ubiquitin E3 ligase. The two elements are linked by spacer groups (linkers) to achieve functional synergy. Common E3 ligases recruited by PROTACs include von Hippel–Lindau (VHL), cereblon (CRBN), and MDM2. When the ternary complex is formed, the E3 ligase catalyzes the transfer of the ubiquitin group to the protein of interest (POI), allowing it to be recognized and degraded by the 26S proteasome (Khan et al., 2020). In addition PROTAC compounds can be recycled in the cell to achieve continuous degradation of novel target proteins. To date, more and more PROTACs for gastrointestinal cancer have been successfully developed, and most of them have been validated as clinical drug targets.

In CRC, xStAx, A1874, dBET1, PROTAC 4, and PROTAC-21a induce degradation of β-catenin, bromodomain-containing protein 4 (BRD4), histone deacetylases (HDACs), and programmed death ligand 1 (PD-L1), block Wnt/MYC signaling, inhibit tumor growth, and restore antitumor immunity (Liao et al., 2020; Qin et al., 2020; Otto et al., 2019; Smalley et al., 2020; Wang Y. et al., 2021). In HCC, BETd-260 and TD-165 trigger BRD4 or CRBN degradation, which promotes mitochondrial dysfunction and apoptosis (Park et al., 2021; Zhang H. et al., 2019). In pancreatic cancer, a variety of PROTACs, such as DX2-145, DP-C series, PRTC and DT2216, exert anti-tumor effects by degrading GRP78, epidermal growth factor receptor (EGFR)/poly(ADP-ribose) polymerase (PARP), and B-cell lymphoma-extra large (BCL-xL), respectively, to overcome drug resistance and inhibit proliferation and invasion (Samanta et al., 2021; Zheng et al., 2021; Ma et al., 2020; Saraswat et al., 2020). In GC, ARV-825 degraded BRD4 more efficiently than conventional inhibitors, downregulated c-Myc, and induced apoptosis and cell cycle arrest (Liao et al., 2021). In ESCC, Src homology 2 domain-containing phosphatase 2 (SHP2)-D26 degrades SHP2 and inhibits extracellular signal-regulated kinase (ERK) signaling, thereby inhibiting tumor cell proliferation (Wang M. et al., 2020) (Table 2). In conclusion, PROTACs represent a promising therapeutic strategy for gastrointestinal cancers by degrading oncoproteins rather than inhibiting them, overcoming drug resistance, and targeting previously untreatable molecules.

**TABLE 2:** Application of PROTACs in digestive system tumors.

Tumor types	PROTACs	E3 ligase	Target protein	References
Colorectal cancer	xStAx	VHL	β-catenin	Liao et al. (2020)
	A1874	MDM2	BRD4	Qin et al. (2020)
	dBET1	CRBN	BRD4	Otto et al. (2019)
	PROTAC 4	CRBN	HDACs	Smalley et al. (2020)
	PROTAC-21a	CRBN	PD-L1	Wang et al. (2021b)
Liver cancer	TD-165	VHL and CRBN	CRBN	Park et al. (2021)
	BETd-260	CRBN	BRD4	Zhang H. et al. (2019)
Pancreatic cancer	DX2-145	CRBN	GRP78	Samanta et al. (2021)
	DP-C	CRBN	EGFR and PARP	Zheng et al. (2021)
	PRTC	VHL	CREPT	Ma et al. (2020)
	DT2216	VHL	BCL-xL	Saraswat et al. (2020)
Gastric cancer	ARV-825	CRBN	BRD	Liao et al. (2021)
Esophageal carcinoma	SHP2-D26	VHL	SHP2	Wang M. et al. (2020)

## 6 Conclusions and future directions

In recent years, ubiquitination has been shown to play a crucial role in tumor development. Significant advances have been made in studying ubiquitination-related pathways and in developing small-molecule inhibitors. However, drugs targeting RING-type E3 ligases are still in the preclinical or clinical research phases. Therefore, one of the main goals of our future research is to better understand the biological roles of E3 ligases in various cancer types and to develop more effective and less toxic small-molecule targeted therapies. By combining these agents with existing treatments such as radiotherapy, chemotherapy, immunotherapy, and cell-based therapies, we aim to improve the clinical effectiveness of combination strategies. Additionally, we will focus on exploring the role of E3 ligases in clinical diagnostics to discover new biomarkers for tumor diagnosis. In summary, an in-depth investigation into the functions of RING-type E3 ligases is expected to open new pathways for the clinical diagnosis and assessment of treatment responses in digestive system tumors and other cancers, thereby enhancing diagnostic accuracy and cure rates, and ultimately advancing clinical translation.

## Glossary

PTMs: Post-translational protein modifications
UPS: Ubiquitin-proteasome system
RING: Really interesting new gene
HECT: Homologous to E6-AP carboxyl terminus
RBR: Ring between ring fingers
IBR: In-between-RING
TRIM: Tripartite motifs proteins
RNF: RING finger protein family
TRAF: TNF receptor-associated factors
cIAP: Cellular inhibitor of apoptosis protein
XIAP: X-linked inhibitor of apoptosis protein
MDM2/4: Murine double minute 2/4
BRCA1: Breast cancer type 1 susceptibility protein
BARD1: Beast cancer-associated RING domain protein 1
CRL: Cullin ring finger ligases
APC/C: Anaphase-promoting complex/cyclosome
MAPK: Mitogen activated protein kinase
SCF: S-phase kinase-associated protein 1–cullin1–F-box protein
SKP1: S-phase kinase-associated protein 1
RBX1: RING-box protein 1
DNA: Deoxyribonucleic acid
EDARADD: EDAR-associated death domain
Snail1: Snail transcriptional repressor 1
EMT: Epithelial-mesenchymal transition
CRC: Colorectal cancer
SMAD4: Decapentaplegic homolog 4
CCL15: C-C motif chemokine ligand 15
CCR1: C-C motif chemokine receptor 1
MST2: STE20-like protein kinase 2
YAP: Yes-associated protein
IRF4: Interferon regulatory factor 4
TLE3: Transducin-like enhancer protein 3
SHP-1: Src homology 2 domain-containing protein tyrosine phosphatase 1
OS: Overall survival
CHAC2: Cation transport regulator-like protein 2
SPHK1: Sphingosine kinase 1
ULK1: Unc-51 like kinase 1
MCPIP1: Monocyte chemoactive protein induced protein 1
LATS2: Large tumor suppressor 2
GSTP1: Glutathione S-transferase pi-1
HCC: Hepatocellular carcinoma
HNF1α: Hepatocyte nuclear factor 1α
MST1: Mammalian STE20-like protein kinase 1
TSC1: Tuberous sclerosis complex 1
CDO1: Cysteine dioxygenase type 1
MTA1: Metastasis-associated protein 1
FoxA1: Forkhead box protein A1
TEAD: Transcriptional enhancer factor domain
NLRP3: NOD-like receptor protein 3
MYL6B: Myosin light chain 6B
HSP90AA1: Heat shock protein 90 alpha A1
KLF4: Krüppel-like factor 4
APOA1: Apolipoprotein A1
LDLR: Low density lipoprotein receiver
IGF2BP2: Insulin like growth factor 2 mRNA binding protein 2
TLR4: Toll like Receptor 4
NAP1L5: Nucleosome assembly protein1-like 5
PHLPP1: PH domain leucine-rich repeat protein phosphatase 1
FBP1: Fructose-1,6-biphosphatase
MMP-9: Matrix metalloproteinase-9
MARCH8: Membrane-associated Ring-CH-type finger 8
PTPN4: Protein tyrosine phosphatase non-receiver type 4
STAT3: Signal transducer and activator of transcription 3
METTL3: Methyltransferase-like 3
SIRT2: Sirtuin 2
eEF1A1: Eukaryotic translation elongation factor 1 alpha 1
GC: Gastric cancer
BAX: Bcl-2 associated X protein
CYLD: Cylindromatosis
SLC7A11: Solute carrier family 7 member 11
GPX4: Glutathione peroxidase 4
MMP2: Matrix metalloprotease-2
PGK1: Phosphoglycerate kinase 1
FOXM1: Forkhead Box M1
RHOC: Ras homolog gene family member C
HDAC1: Histone deacetylase 1
PCDH10: Protocadherin 10
DNMT1: DNA methyltransferase 1
ESCC: Esophageal squamous cell carcinoma
L1CAM: L1 cell adhesion molecule
KLF12: Krüppel-like factor 12
STAT1: Signal transducer and activator of transcription 1
NOTCH2: Notch receptor 2
5-FU: 5-Fluorouracil
ERK2: Extracellular signal-regulated kinase 2
EZH2: Zeste homolog 2
MT2A: Metallothionein 2A
NgBR: Nogo-B receptor
TAK1: Transforming growth factor-β-activated kinase 1
CHD3: Chromodomain helicase DNA-binding protein 3
CDC20: Cell division cycle protein 20
CDH1: CDC20 homolog 1
TAME: N-4-Tosyl-L-arginine methyl ester
PROTAC: Proteolysis targeting chimera
VHL: Von Hippel–Lindau
CRBN: Cereblon
POI: Protein of interest
BRD4: Bromodomain-containing protein 4
HDACs: Histone deacetylases
PD-L1: Programmed death ligand 1
EGFR: Epidermal growth factor receptor
PARP: Poly(ADP-ribose) polymerase
BCL-xL: B-cell lymphoma-extra large
SHP2: Src homology 2 domain-containing phosphatase 2
ERK: Extracellular signal-regulated kinase
