# Supplementary material for: Ubiquitination mediated by RING-type E3 ligases in the progression of digestive system tumors: mechanistic insights and potential therapeutic strategies
Source: Front Cell Dev Biol. 2026 May 20;14:1809222. doi: 10.3389/fcell.2026.1809222 (PMC13230128; doi:10.3389/fcell.2026.1809222)
Supplement: Supplementary file 1 [file Table1.docx]

***Supplementary Table 1*** *Regulatory mechanisms, functions and Clinical applications of RING-type E3 ligases in digestive system tumors.*

| ***E3 Ligase Subfamilies*** | ***RING-type E3 ligases*** | ***Tumor types*** | ***Related mechanisms*** | ***Functions*** | ***Experimental model systems*** | ***Clinical application(s)*** | ***Reference*** |
| --- | --- | --- | --- | --- | --- | --- | --- |
| ***TRIM Family*** | *TRIM7* | *Gastric Cancer* | *TRIM7-SLC7A11-GPX 4* | *Inhibit proliferation.* | *GC cell lines (AGS, HGC27), 38 pairs of GC and matched adjacent tissues, tissue microarray and BALB/c nude mice (6-week-old) used for the xenograft tumor modeling* | *Down-regulation, poor prognosis* | *(Chen et al., 2024c)* |
|  | *TRIM8* | *Liver Cancer* | *TRIM8-HNF1α* | *Promote proliferation, colony formation, invasion and migration.* | *Human hepatocellular carcinoma cell lines including Huh7, HepG2, MHCC-L, Hep3B, PLC and SK-Hep1 for in vitro assays, clinical HCC and paired adjacent non-tumor tissues for ex vivo validation, and BALB/c nude mouse subcutaneous xenograft model for in vivo tumor verification* | *Up-regulation, poor prognosis* | *(Peng et al., 2024)* |
|  | *TRIM15* | *Pancreatic Cancer* | *TRIM15-APOA1-LDLR* | *Promote invasion, migration and metastasis.* | *pancreatic cancer cell lines (PANC-1 and BxPC-3), clinical pancreatic cancer patient specimens (ex vivo validation), BALB/c nude mice metastasis model (in vivo model)* | *Up-regulation, poor prognosis* | *(Sun et al., 2021)* |
|  | *TRIM15* | *Pancreatic Cancer* | *TRIM15-IGF2BP2-TLR4* | *Promote colony formation, proliferation, migration and invasion.* | *pancreatic cancer cell lines ( PANC-1, BxPC-3 and Panc02 ), clinical pancreatic cancer patient specimens (ex vivo validation), C57bl/6 mice xenograft model (in vivo model)* |  | *(Cai et al., 2024a)* |
|  | *TRIM16* | *Colorectal Cancer* | *TRIM16-Snail1* | *Inhibit EMT, migration, invasion, and metastasis.* | *Colorectal cancer cell lines (e.g., HCT116, SW480, and LoVo) for in vitro functional assays, clinical CRC specimens for ex vivo analysis, and BALB/c nude mouse tail vein injection lung metastasis model for in vivo assessment* | *Down-regulation, poor prognosis* | *(Ruan et al., 2021)* |
|  | *TRIM17* | *Gastric Cancer* | *TRIM17-BAX* | *Promote proliferation.* | *GC cell lines (AGS, HGC-27, MKN45, NCI-N87), 5-week-old female NOD/SCID mice, 6- to 8-week-old female BALB/c nude mice, and human GC tissue microarray* | *Up-regulation, poor prognosis* | *(Shen et al., 2023)* |
|  | *TRIM21* | *Colorectal Cancer* | *EDARADD-PPARa-Trim21-Snail1* | *Promote proliferation and EMT.* | *colon cancer cell lines (in vitro model) and mouse tumor xenograft models (in vivo model)* |  | *(Yang et al., 2023)* |
|  | *TRIM21* | *Colorectal Cancer* | *TRIM21-MST2-YAP* | *Inhibit invasion and metastasis.* | *Human colon cell lines ( RKO, HCT116, SW480, and SW620) , C57BL/6 background Trim21 knockout mice and immunodeficient BALB/c nude mice, and tumor organoids* | *Down-regulation, poor prognosis* | *(Liu et al., 2023)* |
|  | *TRIM21* | *Liver Cancer* | *TRIM21-MST1-YAP* | *Promote proliferation and migration.* | *Human hepatocellular carcinoma cell lines Huh7, SK-Hep1 and Hep3B for in vitro functional assays, clinical HCC specimens for ex vivo analysis, and BALB/c nude mouse tail vein injection lung metastasis model for in vivo assessment* | *Up-regulation, poor prognosis* | *(Shu et al., 2024)* |
|  | *TRIM25* | *Liver Cancer* | *TRIM25-MTA1* | *Inhibit migration and invasion.* | *Human hepatocellular carcinoma cell line HuH6 and normal hepatic cell line THLE-2 for in vitro functional assays, The Cancer Genome Atlas data from 25 HCC patients for ex vivo analysis, and BALB/c nude mouse subcutaneous xenograft model using Huh7 cells for in vivo assessment* | *Down-regulation* | *(Zang et al., 2017)* |
|  | *TRIM25* | *Gastric Cancer* | *JP3-TRIM25-SP1-MMP2* | *Inhibit angiogenesis, proliferation and metastasis.* |  | *Down-regulation, poor prognosis* | *(Chen et al., 2020)* |
|  | *TRIM27* | *Esophageal Cancer* | *TRIM27-KLF12-L1CAM* | *Promote cisplatin resistance and tumor metastasis.* | *Human ESCC cell lines (KYSE30, KYSE70, KYSE150, KYSE180, KYSE450, KYSE510), human NSCLC cell line H1299, human glioblastoma cell line U87, human hepatocellular carcinoma cell line HepG2, normal human embryonic kidney cell line HEK293, patient-derived xenografts (5-week-old BALB/c nude mice), cell line-derived tumor xenografts (BALB/c nude mice)* |  | *(Zhang et al., 2024a)* |
|  | *TRIM28* | *Liver Cancer* | *UBE2S-TRIM28-p27* | *Promote proliferation, invasion, migration and metastasis.* | *Human hepatocellular carcinoma cell lines Huh-7, HepG2 and MHCC-97H for in vitro experiments, clinical HCC and adjacent normal tissues for ex vivo validation, and BALB/c nude mouse subcutaneous xenograft plus orthotopic liver tumor models for in vivo evaluation* | *Up-regulation, poor prognosis* | *(Zhang et al., 2021)* |
|  | *TRIM29* | *Pancreatic Cancer* | *NAP1L5-TRIM29-PHLPP1-AKT/mTOR* | *Promote proliferation, migration, and invasion.* | *pancreatic ductal adenocarcinoma cell lines (PANC-1, Mia PaCa-2, SW1990; in vitro model), clinical PDAC patient tissue specimens (ex vivo validation), nude mouse subcutaneous xenograft model (in vivo model)* |  | *(Xiao et al., 2023)* |
|  | *TRIM31* | *Liver Cancer* | *TRIM31-(TSC1-TSC2)-mTORC1* | *Promote proliferation, invasion and colony formation.* | *Human hepatocellular carcinoma cell lines for in vitro functional and mechanistic assays, clinical HCC and paired non-cancerous liver tissues for ex vivo expression analysis, and nude mouse subcutaneous xenograft model for in vivo validation* | *Up-regulation, poor prognosis* | *(Guo et al., 2018)* |
|  | *TRIM33* | *Esophageal Cancer* | *TRIM33-p53* | *Enhance aerobic glycolysis and promote proliferation.* | *ESCC cell lines (Eca109, KYSE150, TE-1), 4- to 6-week-old female BALB/c nude mice, and ESCC patient tissues with normal esophageal tissues* | *Up-regulation, poor prognosis* | *(Xia et al., 2024)* |
|  | *TRIM47* | *Colorectal Cancer* | *TRIM47-SMAD4-CCL15/CCR1* | *Promote proliferation, invasion and metastasis.* | *Colorectal cancer cell lines (HCT116, HT29, SW480, RKO, SW620, Caco2, LoVo, and SW1116) for in vitro functional assays, clinical CRC specimens (100 fresh tissues and 180 formalin-fixed paraffin-embedded tissues from Renji Hospital) for ex vivo analysis, and BALB/c nude mouse xenograft models (subcutaneous tumor growth and tail vein injection lung metastasis models) for in vivo assessment* | *Up-regulatio**n, poor prognosis* | *(Liang et al., 2019)* |
|  | *TRIM47* | *Liver Cancer* | *TRIM47-CDO1* | *Promote proliferation, migration and invasion.* | *Human hepatocellular carcinoma cell lines for in vitro ferroptosis and proliferation assays, clinical HCC tissues from TCGA cohort and patient samples for ex vivo validation, and immunodeficient mouse subcutaneous xenograft model for in vivo verification* | *Up-regulation, poor prognosis* | *(Zhang et al., 2024b; Liang et al., 2019)* |
|  | *TRIM47* | *Pancreatic Cancer* | *TRIM47-FBP1* | *Promote aerobic glycolysis and proliferation.* | *pancreatic cancer cell lines (e.g., PANC‑1, Mia PaCa‑2; in vitro model), clinical pancreatic cancer patient specimens (ex vivo validation), nude mouse subcutaneous xenograft & lung metastasis model (in vivo model)* | *Up-regulation, poor prognosis* | *(Li et al., 2021)* |
|  | *TRIM47* | *Gastric Cancer* | *TRIM47-CYLD-NF-κB* | *Promote proliferation, invasion, migration.* | *gastric cancer cell lines (AGS, HGC-27), normal gastric mucosal cell line GES-1, 20 pairs of gastric cancer and adjacent tissue samples (from Renmin Hospital of Wuhan University), 4-week-old female BALB/c nude mice* | *Up-regulation, poor prognosis* | *(Wang et al., 2024)* |
|  | *TRIM50* | *Liver Cancer* | *TRIM50-SNAIL* | *Inhibit colony formation, proliferation, invasion, EMT and metastasis.* | *Human hepatocellular carcinoma cell lines BEL7402, HUH7, HepG2 and SMMC7721 for in vitro functional assays, clinical HCC and paired non-cancerous liver tissues for ex vivo validation, and BALB/c nude mouse subcutaneous xenograft model for in vivo antitumor assessment* | *Down-regulation, poor prognosis* | *(Ma et al., 2018)* |
|  | *TRIM50* | *Gastric Cancer* | *TRIM50-JUP-c-MYC* | *Inhibit proliferation, migration and metastasis.* | *GC cell lines (MKN7, MKN74, AGS, MKN45, SNU-668, HGC27) purchased from ATCC, 4- to 6-week-old female BALB/c nude mice, gastric cancer and normal gastric tissues, human GC tissue microarray* | *Down-regulation, poor prognosis* | *(Hu et al., 2023)* |
|  | *TRIM50* | *Gastric Cancer* | *TRIM50-PGK1* | *Inhibit glycolytic, proliferation, migration and invasion.* | *GC patient tumor tissues, adjacent nontumor tissues, GES-1 human gastric epithelial cell line, GC cell lines (HGC-27, MKN-45, AGS, NCI-N87) and mouse lung/liver metastasis model* | *Down-regulation, poor prognosis* | *(Gu et al., 2024)* |
|  | *TRIM54* | *Gastric Cancer* | *TRIM54-Filamin C* | *Promote proliferation, invasion, migration.* | *pancreatic cancer cell lines, paired pancreatic cancer tumor and adjacent non-tumor tissues, male athymic nude mice* | *Up-regulation, poor prognosis* | *(Cao et al., 2022)* |
|  | *TRIM58* | *Gastric Cancer* | *TRIM58-β-catenin* | *Inhibit proliferation.* | *Cancer and normal tissue samples, GC cell lines (MKN45, BGC823, HGC27, AGS, SNU719), gastric mucosa cell line GES-1and nude mice* | *Down-regulation* | *(Liu et al., 2020)* |
|  | *TRIM59* | *Gastric Cancer* | *TRIM59-p53* | *Promote proliferation and migration.* | *GC cell lines ( MKN45, AGS, SGC7901, BGC823, Snu5, N87, and Snu1), xenograft tumors in nude mice and human GC tissue microarray* | *Up-regulation, poor prognosis* | *(Zhou et al., 2014)* |
|  | *TRIM65* | *Colorectal Cancer* | *TRIM65-ARHGAP35* | *Promote proliferation, migration, invasion and metastasis to the liver and lungs of CRC.* | *Human colon cell lines (HT29, DLD-1, RKO, LOVO, HCT15, HCT116) and mouse colon cancer cell lines (CT26, MC38) , female BALB/c nude mice, and human clinical specimens( ex vivo)* | *Up-regulation, poor prognosis* | *(Chen et al., 2019)* |
| ***RNF Family*** | *RING1* | *Liver Cancer* | *RING1-p53* | *Promote proliferation, migration and metastasis.* | *Human hepatocellular carcinoma cell line HepG2 and colorectal cancer cell line HCT116 for in vitro assays,* *clinical HCC tissues for ex vivo validation, and BALB/c nude mouse subcutaneous xenograft model for in vivo tumor growth evaluation* | *Up-regulation, poor prognosis* | *(Shen et al., 2018)* |
|  | *RNF2* | *Colorectal Cancer* | *RNF2-IRF4* | *Promote proliferation, migration and invasion.* | *colon cancer cell lines (SW480 and HCT116) (in vitro model) , BALB/c nude male mice and clinical CRC tissues* | *Up-regulation, poor prognosis* | *(Wang et al., 2022)* |
|  | *RNF6* | *Colorectal Cancer* | *RNF6-TLE3-Wnt/β-catenin* | *Promote proliferation, EMT, migration, invasion and metastasis.* | *colon cancer cell lines (DLD-1, HCT116, HT29, LOVO, SW480, SW620, and SW1116) , colorectal cancer tumor and adjacent nontumor tissue samples and male athymic Balb/c nude mice* | *Up-regulation, poor prognosis* | *(Liu et al., 2018b)* |
|  | *RNF6* | *Colorectal Cancer* | *RNF6-SHP-1-JAK/STAT3* | *Promote proliferation, invasion and metastasis.* | *colorectal cancer cell lines ( RKO, SW1116, SW480, Caco2, LoVo, HT29, and HCT116 ), male BALB/c nude mice (in vivo model), and clinical CRC patient samples (ex vivo validation)* | *Up-regulation, poor prognosis* | *(Liang et al., 2018)* |
|  | *RNF6* | *Liver Cancer* | *RNF6-FoxA1* | *Promote metastasis, EMT and enhance radioresistance.* | *Human hepatocellular carcinoma cell lines for in vitro metastasis and radioresistance assays, clinical HCC specimens for ex vivo validation, and nude mouse xenograft and pulmonary metastasis models for in vivo assessment* | *Up-regulation, poor prognosis* | *(Cai et al., 2019)* |
|  | *RNF8* | *Colorectal Cancer* | *RNF8-β-catenin-c-Myc* | *Promote proliferation.* | *colon cancer cell line ( HCT116 ) and xenograft mouse models ( BALB/C, 4-weeks-old, female )* | *Up-regulation, poor prognosis* | *(Ren et al., 2020)* |
|  | *RNF13* | *Pancreatic Cancer* | *RNF13-MMP-9* | *Promote invasion.* | *Human pancreatic cancer cell lines( MiaPaca-2, COS-7 cells) for in vitro molecular mechanism exploration and clinical pancreatic cancer patient tissue samples for ex vivo expression profiling* | *Up-regulation, correlate with histological grading* | *(Zhang et al., 2009)* |
|  | *RNF20* | *Liver Cancer* | *RNF20-NLRP3* | *Inhibit proliferation and metastasis.* | *Serum samples from liver cancer patients for ex vivo analysis, liver cancer cell lines for in vitro functional assays* | *Down-regulation, poor prognosis* | *(Liu et al., 2024a)* |
|  | *RNF38* | *Gastric Cancer* | *RNF38-SHP-1-STAT3* | *Promote proliferation.* | *GC cell lines (AGS, NCI-N87, MGC80-3, SNU-1), human normal gastric mucosal cell line GES-1, primary gastric cancer tissues, paracancerous normal tissues and nude mice* | *Up-regulation, poor prognosis* | *(Zhang et al., 2018)* |
|  | *RNF43* | *Pancreatic Cancer* | *RNF43-BRAF/MEK* | *Inhibit proliferation.* | *Pancreatic cancer cell lines (PANC-1, AsPC-1, HPAF-II, CaCO2, SW48), and Advanced Severe Immuno Deficiency (ASID) mice* |  | *(Hsu et al., 2024)* |
|  | *RNF112* | *Gastric Cancer* | *RNF112-FOXM1* | *Inhibit proliferation and invasion.* | *GC cell lines (BGC823, MGC803) and nude mice* | *Down-regulation, poor prognosis* | *(Zhang et al., 2023b)* |
|  | *RNF126* | *Colorectal Cancer* | *RNF126-p53* | *Promote proliferation, migration and drug resistance.* | *colorectal cancer cell lines (HCT116, HCT-8, Colo205, and SW620) (in vitro model), clinical CRC patient specimens (ex vivo validation)* | *Up-regulation, poor prognosis* | *(Wang et al., 2020b)* |
|  | *RNF148* | *Colorectal Cancer* | *RNF148-CHAC2* | *Promote proliferation and migration, induces acquired resistance.* | *colorectal cancer cell lines ( COLO320, DLD1, SW620, RKO, HCT116, LoVo, HT29 and SW48), clinical CRC patient specimens (ex vivo validation), and xenograft mouse models ( female BALB/c nude mice)* | *Up-regulation, poor prognosis* | *(Liu et al., 2024b)* |
|  | *MARCH5(RNF153)* | *Liver Cancer* | *MARCH5-p53* | *Promote proliferation, invasion, migration and metastasis.* | *Human hepatocellular carcinoma cell lines( Hep3B, LM3, Huh7, HepG2, MHCC97H) for in vitro proliferation and ubiquitination assays, clinical HCC and adjacent normal tissues for ex vivo validation, and 5-week-old female BALB/c nude mice for subcutaneous xenograft model* | *Up-regulation, poor prognosis* | *(Cai et al., 2024b)* |
|  | *DTX3 (RNF154)* | *Esophageal Cancer* | *DTX3-NOTCH2* | *Inhibit proliferation and migration.* | *Normal human esophageal epithelial cell line HEEC, ESCC cell lines (KYSE150, TE-1, Eca-109) and nude mice* | *Down-regulation* | *(Ding et al., 2020)* |
|  | *RNF168* | *Gastric Cancer* | *RNF168-RHOC-HDAC1* | *Inhibit proliferation, invasion, migration.* | *GC cell lines (SGC-7901, BGC-823), male BALB/c nude mice and GC tissues with matched non-tumor tissues* | *Down-regulation, poor prognosis* | *(Xu et al., 2021)* |
|  | *RNF168* | *Esophageal Cancer* | *RNF168-JAK/STAT1* | *Promote proliferation and invasion.* | *ESCC cell lines (NEC, EC109) and ESCC patient tissues* | *Up-regulation, poor prognosis* | *(Yu et al., 2019)* |
|  | *MARCH8 (RNF178)* | *Pancreatic Cancer* | *MARCH8-PTPN4-STAT3* | *Promote proliferation, migration and invasion of pancreatic cancer cells, and inhibit cell apoptosis.* | *Human pancreatic cancer cell lines for in vitro functional assays, clinical pancreatic cancer specimens for ex vivo analysis, and mouse models for in vivo assessment.* |  | *(Chen et al., 2023)* |
|  | *RNF 180* | *Gastric Cancer* | *RNF180-DNMT1-PCDH10* | *Inhibit proliferation, invasion, migration and metastasis.* | *142 paraffin-embedded advanced gastric adenocarcinoma (pT2–4NanyM0) specimens and paired adjacent noncancerous tissues, stable GC cell lines and LV-PCDH10-transfected HGC27 cells were injected in the tail vein of nude mice* | *Down-regulation, poor prognosis* | *(Zhang et al., 2023a)* |
|  | *RNF185* | *Gastric Cancer* | *RNF185-JWA* | *Promote migration and metastasis.* | *GC cell lines (NCI-N87, HGC27, SGC7901, BGC823, MGC803), GES-1 cell line, samples from GC patients and six-week-old BALB/c nude mice* | *Up-regulation, poor prognosis* | *(Qiu et al., 2018)* |
|  | *RNF214* | *Liver Cancer* | *RNF214-TEAD-YAP* | *Promote proliferation, invasion, migration and inhibit apoptosis.* | *Human hepatocellular carcinoma cell lines ( HLF、HLE、HepG2、Huh7、Hep3b、Huh1、MEF )for in vitro signaling and functional assays, clinical HCC tissues for ex vivo validation, and 5-week-old nude mice for in vivo tumor progression evaluation* | *Up-regulation, poor prognosis* | *(Lin et al., 2024)* |
| ***TRAF Family*** | *TRAF6* | *Colorectal Cancer* | *SPHK1-TRAF6-ULK1* | *Promote proliferation, invasion and metastasis.* | *colorectal cancer cell lines (RKO, HT29) (in vitro model) and clinical CRC patient specimens (tumor tissues and corresponding normal tissues )* |  | *(Chen et al., 2024a)* |
|  | *TRAF6* | *Colorectal Cancer* | *LPS-TRAF6-NF-κB-VEGF-C* | *Promote proliferation, migration, invasion and lymphatic metastasis.* | *colorectal cancer cell lines (SW480 and HCT116), clinical CRC patient specimens ( human colorectal tumor tissue and adjacent corresponding non‐tumor tissue samples), and* *xenograft mouse models ( female BALB/c nude mice )* |  | *(Guangwei et al., 2022)* |
|  | *TRAF6* | *Colorectal Cancer* | *MCPIP1-TRAF6-NF-κ B* | *Inhibit proliferation and migration.* | *colorectal cancer cell lines (HCT116, SW480), clinical CRC patient specimens and* *male* *athymic BALB/c nude mice* |  | *(Ye et al., 2023)* |
|  | *TRAF6* | *Colorectal Cancer* | *TRAF6-β-catenin* | *Inhibit invasion and metastasis.* | *colorectal cancer cell lines HCT116, SW480, HT-29, DLD-1 , RKO), clinical CRC patient specimens (ex vivo validation) and male athymic BALB/c nude mice* |  | *(Wu et al., 2019)* |
| ***MDM Family*** | *MDM2* | *Liver Cancer* | *MYL6B-MDM2-p53* | *Promote proliferation and inhibit cell apoptosis.* | *Human hepatocellular carcinoma cell lines( Huh7 and SK-HEP-1) for in vitro functional and ubiquitination assays* |  | *(Xie et al., 2018)* |
|  | *MDM2* | *Liver Cancer* | *gp96-MDM2-p53* | *Promote proliferation.* | *Human hepatocellular carcinoma cell lines(SK-Hep-1 and HepG2) for in vitro p53 stability and E3 ligase activity assays and nude mouse xenograft model for in vivo verification* |  | *(Wu et al., 2015a)* |
| ***SCF-type E3 Ligases*** | *FBXL2* | *Gastric Cancer* | *FBXL2-FoxM1* | *Inhibit proliferation and invasion.* | *15 paired gastric cancer and adjacent nontumor normal tissues and GC cell line NCI-N87* | *Down-regulation* | *(Li et al., 2016)* |
|  | *FBXL5* | *Gastric Cancer* | *FBXL5-Snail1* | *Inhibit invasion and migration.* | *Co-immunoprecipitation performed in GC cells and verified their relationship in GC patient samples* | *Down-regulation* | *(Wu et al., 2015b)* |
|  | *FBXL6* | *Liv**er Cancer* | *FBXL6-HSP90AA1-c-MYC* | *Promote proliferation.* | *Human hepatocellular carcinoma cell lines Huh3B and SMMC-7721 for in vitro proliferation, ubiquitination and oncogenic functional assays, paired clinical HCC and adjacent non-tumor liver specimens for ex vivo biomarker expression verification, and BALB/c nude mouse subcutaneous xenograft in vivo model for tumor growth evaluation* | *Up-regulation* | *(Shi et al., 2020)* |
|  | *FBXL8* | *Colorectal Cancer* | *FBXL8-TP53* | *Promote proliferation, migration, invasion, liver metastasis and stem cell characteristics.* | *colorectal cancer cell lines HT29, HCT116, SW48 and HCT15), clinical CRC patient specimens and male BABL/C female nude mice* | *Up-regulation, poor prognosis* | *(Yao et al., 2023)* |
|  | *FBXL19* | *Esophageal Cancer* | *FBXL19-Rac3-TGFβ1-E-cadherin* | *Promote migration, invasion and EMT.* | *Esophageal adenocarcinoma (OE19 and OE33) cancer cells* |  | *(Dong et al., 2014)* |
|  | *FBXO22* | *Liver Cancer* | *FBXO22-p21* | *Promote proliferation.* | *Human hepatocellular carcinoma cell lines HL-7702, HepG2, Huh7, Hep3B, Bel-7402, HLF, LM3 for in vitro cell cycle, matched human HCC clinical tissues and peritumoral samples for ex vivo correlation analysis, and BALB/c nude mouse subcutaneous xenograft in vivo tumorigenesis model for phenotypic validation* | *Up-regulation, poor prognosis* | *(Zhang et al., 2019b)* |
|  | *FBXO22* | *Liver Cancer* | *FBXO22-**KLF4* | *Promote proliferation.* | *Human hepatocellular carcinoma cell lines HepG2, HuH7 and Hep3B for in vitro KLF4-mediated ubiquitination, migration and invasion functional experiments, clinical HCC patient tissue cohorts for ex vivo pathological expression profiling, and male BALB/c nude mice subcutaneous xenograft in vivo model for malignant progression assessment* | *Up-regulation, poor prognosis* | *(Tian et al., 2015)* |
|  | *FBXO31* | *Pancreatic Cancer* | *METTL3-FBXO31-SIRT2* | *Promote proliferation, migration and invasion.* | *Pancreatic cancer cell lines (Patu-8988, Panc-1), five-weeks-old female nude mice, and human pancreatic cancer microarray slides (HpanA150su01)* | *Up-regulation, poor prognosis* | *(Chen et al., 2024b)* |
|  | *FBXO32* | *Pancreatic Cancer* | *FBXO32-eEF1A1* | *Promoting migration, invasion, and metastasis.* | *Pancreatic cancer cell lines, human pancreatic cancer and adjacent nontumor tissue samples, and female athymic nude mice for in vivo tumor growth and metastasis models* | *Up-regulation, poor prognosis* | *(Su et al., 2024)* |
|  | *FBXW10* | *Colorectal Cancer* | *FBXW10-LATS2* | *Promote proliferation, migration, angiogenesis and liver metastasis.* | *colorectal cancer cell lines ( HCT116, SW480), clinical CRC patient specimens (ex vivo validation), nude mouse liver metastasis & angiogenesis model (in vivo model)* | *Up-regulation, poor prognosis* | *(Zhang et al., 2023d)* |
|  | *FBX8* | *Colorectal Cancer* | *FBX8-GSTP1* | *Inhibit proliferation, invasion and metastasis.* | *Classical colorectal cancer cell lines including LoVo, SW480， HCT116 and SW620 for in vitro ubiquitination and tumor suppressive assays, paired human colorectal clinical specimens for ex vivo biomarker verification, and C57BL/6 mice* |  | *(FeiFei et al., 2019)* |
